# Development and Characterization of Chitosan Microparticles via Ionic Gelation for Drug Delivery

**DOI:** 10.3390/polym17192603

**Published:** 2025-09-26

**Authors:** Zahra Rajabimashhadi, Annalia Masi, Sonia Bagheri, Claudio Mele, Gianpiero Colangelo, Federica Paladini, Mauro Pollini

**Affiliations:** 1 Department of Innovation Engineering, University of Salento, 73100 Lecce, Italy; annalia.masi@unisalento.it (A.M.); sonia.bagheri@unisalento.it (S.B.); claudio.mele@unisalento.it (C.M.); gianpiero.colangelo@unisalento.it (G.C.); 2 Department of Experimental Medicine (DiMeS), University of Salento, 73100 Lecce, Italy; mauro.pollini@unisalento.it

**Keywords:** chitosan microparticles, ionic gelation, drug delivery, controlled release

## Abstract

This study explores the formulation of chitosan microparticles through ionic gelation and presents detailed physicochemical characterization, release studies, and the utility and potential uses for drug delivery. Three formulations were prepared under rate-controlled conditions (stirring at 800 rpm and pH maintained at 4.6) with and without stabilizers to examine the effects of formulation parameters on particle morphology and structural stability. To determine different structural and chemical characteristics, Attenuated Total Reflectance Fourier-Transform Infrared spectroscopy (ATR–FTIR), Scanning Electron Microscopy (SEM), and dynamic light scattering (DLS) were utilized, which confirmed that the particles formed and assessed size distribution and structural integrity. Atomic force microscopy (AFM) was used to quantify surface roughness and potential nanomechanical differences that may derive from the use of different modifiers. Coformulation of bovine serum albumin (BSA) permitted assessment of encapsulation efficiency and drug release capacity. Based on in vitro release evidence, the protein released at a different rate, and the dispersion of formulations under physiological conditions (PBS, pH 7.4, 37 °C) confirmed the differences in stability between formulations. The tunable physical characteristics, mild fabrication conditions, and controlled drug release demonstrated that the chitosan particles could have useful relevance as a substrate for localized drug delivery and as a bioactive scaffold for tissue regenerative purposes.

## 1. Introduction

Common drug delivery systems continue to show limitations associated with poor bioavailability, a lack of targeted specificity, and rapid systemic clearance, which can interfere with therapeutic effectiveness, as well as increase side effects [1,2]. Research has transitioned towards biological materials that can provide a site-specific, sustained and controlled delivery of therapeutics, mostly in the treatment of chronic diseases, post-surgical wound care, and instances when systemic dosing that has to be repeated frequently is ineffective or dangerous [3,4].

Biopolymers are an appealing option in drug delivery because they are inherently biocompatible, biodegradable, and have chemical and physical characteristics that can be modified [5,6]. Chitosan, a cationic polysaccharide obtained from partial deacetylation of chitin from crab and shrimp shells, is among the most explored biopolymers in drug delivery [7,8]. These features of chitosan, including biodegradability, mucoadhesiveness, positive surface charge, and the availability of a reactive amine group, permit ionic crosslinking under mild conditions and the ability to entrap sensitive bioactives (i.e., proteins and peptides) that are structurally modified [9,10,11], with cross-linking representing an important factor to the particle size, surface charge, morphology and protein encapsulation efficacy [12].

Chitosan nanoparticles (CS-NPs) have also been extensively studied and explored for systemic drug delivery; however, systemic (especially rapid clearance) toxicity has reduced the Translational potential for CS-NPs, in particular with other environmental influences. Similarly to hydrogels, hydrophilic chitosan microparticles (CS-MPs) have unique and beneficial properties, including mechanical stability, prolonged release, and limited penetration to tissue sites, making them ideal for localized delivery applications (like wound healing, coatings on implants, and site-specific protein delivery) [13,14]. CS-MPs can be generated through scalable, aqueous ionic gelation, potentially without requiring damaging chemical crosslinkers or detrimental impacts on biomolecules. Various studies have examined PBS-stabilized CS-MPs [15]; however, they did not fully quantify the comparative effects of TPP crosslinking with respect to each particle stability, release kinetics, and associated potential applications. This work describes a systematic comparison of TPP-crosslinked CS-MPs to PBS-stabilized CS-MPs, and the implications relative to drug delivery applications. Chitosan has also been employed as a versatile support in catalytic systems, where ionic interactions with tripolyphosphate (TPP) were shown to stabilize the polymeric structure and allow for functional use [16].

Even with these advantages, research related to degradation patterns and mechanical properties of CS-MPs under physiologically relevant conditions has been limited. Typically, research related to chitosan degradation has focused on hydrogels or nanoparticles. This lack of information can limit our understanding of microparticles [17]. Degradation may significantly affect the release profile, structural integrity of CS-MPs and biocompatibility of CS-MPs, especially when combined with physiologically relevant enzymes like lysozymes or pH. Some mechanical characterization techniques like atomic force microscopy (AFM) could give insight into the stiffness, surface morphology, and structure evolution in CS-MPs during degradation, but these types of studies are rare in the literature and often fail to relate mechanical properties and release profiles.

In this study, we developed and compared three different drug-loaded chitosan microparticle formulations produced via ionic gelation under varying stabilizing conditions (without stabilizer, TPP, and PBS). We used bovine serum albumin (BSA) as a model protein drug, and characterized its physicochemical properties, mechanical properties via AFM, and in vitro release kinetics. The aim of this research is to examine how the formulation parameters related to the mechanical properties and release profiles, which would allow us to better rationally design tunable with predictable performance chitosan microparticles-based delivery systems in clinical protein therapeutics.

## 2. Materials and Methods

### 2.1. Materials

Chitosan (medium molecular weight, 99.9% deacetylated), sodium tripolyphosphate (TPP, 99.9%), bovine serum albumin (BSA), acetic acid (99.9%), and sodium hydroxide (NaOH, 1N) were all purchased from Sigma-Aldrich (St. Louis, MO, USA). Phosphate-buffered saline (PBS, tablet) was also purchased from Sigma-Aldrich (St. Louis, MO, USA) and the solution was prepared in the laboratory. All solutions were made using deionized water (for synthesis) which was purchased from Sigma-Aldrich (St. Louis, MO, USA).

### 2.2. Preparation of Chitosan Microparticles Without Drug Encapsulation

#### 2.2.1. Chitosan Solution Preparation

**Figure 1:**
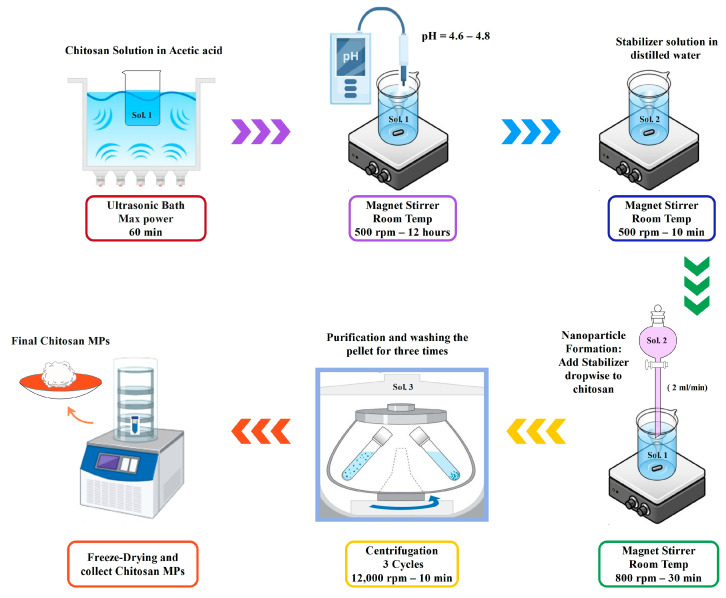
Stepwise Schematic of Chitosan Microparticles Preparation via Ionic Gelation.

A 0.6% (*w*/*v*) chitosan solution was made by dissolving 0.6 g of chitosan in 100 mL of 1% (*v*/*v*) acetic acid with magnetic stirring at 500 rpm for 2–4 h at room temperature, until fully dissolved. The pH of the solution was adjusted to 4.6–4.8 by adding 1N NaOH dropwise and measuring with a calibrated pH meter. The preparation process of chitosan microparticles through ionic gelation is summarized in Figure 1.

#### 2.2.2. TPP Solution Preparation

A 0.3% (*w*/*v*) TPP solution was prepared by dissolving 0.3 g of TPP in 100 mL of DI water and stirring until fully dissolved.

#### 2.2.3. Microparticle Formation via Ionic Gelation

The microparticles were formed by adding the TPP solution dropwise into the chitosan solution under continuous stirring (800 rpm) at room temperature (~25 °C). In PBS-stabilized samples, PBS was also added at the same rate and conditions. Stirring was continued for 30 min after the addition to ensure complete ionic crosslinking. Opalescence in the mixture indicated particle formation.

#### 2.2.4. Purification and Collection

The final suspensions were centrifuged at 10,000 rpm for 30 min at 4 °C. The pellets were washed three times with deionized water to remove unreacted chemicals and freeze-dried for further analysis. Three batches were synthesized with the same rate-controlling conditions but with different treatments of stabilizers to study its effect. Both BSA-loaded and unloaded microparticles were centrifuged under identical conditions (10,000 rpm for 30 min at 4 °C), ensuring that potential effects on yield or aggregation were consistent across all samples. A summary of the characteristics of prepared batches is detailed in Table 1.

### 2.3. Preparation of Chitosan Microparticles with Drug Encapsulation

The process for microparticle synthesis was as described above, except that BSA (30 mg) was introduced directly to the chitosan solution after pH adjustment and before adding the crosslinking or stabilizing agent. The BSA was allowed to stir and dissolve completely for over 30 min. TPP or PBS was dropwise added to the chitosan and BSA after the BSA went into solution to initiate microparticle formation and encapsulate BSA via ionic gelation. The purification and collection processes remained the same as the un-loaded microparticles.

### 2.4. Characterization

#### 2.4.1. Attenuated Total Reflectance Spectroscopy (ATR)

Attenuated Total Reflectance (ATR) spectroscopy was utilized to investigate the chemical groups present on the chitosan microparticles to verify chemical structure and crosslinking interactions. The analysis was completed using an ATR Jasco 6300 spectrometer (JASCO Corporation, Tokyo, Japan). Infrared spectra were recorded in the range of 400 to 4000 cm^−1^ with 128 scans and a resolution of 4 cm^−1^ via a germanium round crystal window.

#### 2.4.2. Scanning Electron Microscopy (SEM)

The morphology of the chitosan microparticles was examined using a SEM EVO^®^ 40 (Carl Zeiss AG, Jena, Germany). Approximately 1–2 mg of the dried microparticles were placed onto a carbon adhesive tape mounted on an aluminum stub. The samples were then observed under an accelerating voltage of 20 kV without sputter coating. Charging artefacts were minimized by careful optimization of imaging parameters, including working distance, spot size, and scan rate. The resulting micrographs showed clear particle morphology with no evidence of distortion or artefacts.

#### 2.4.3. Dynamic Light Scattering (DLS) and Zeta Potential

Hydrodynamic diameter and Zeta potential measurements were carried out using the Malvern Zetasizer Nano ZS (Malvern Panalytical Ltd., Malvern, UK), a device that measures not only particle size (DLS technique), but also zeta potential (Table 2). All DLS and zeta potential measurements were performed at pH 4.6 to ensure comparability across formulations.

In particular, Dynamic light scattering (DLS) is a widely employed technique for determining the size distribution of particles in suspension. Specifically, DLS is utilized to measure the intensity autocorrelation function of the scattered light emitted by particles in suspension. This autocorrelation function provides valuable insights into the Brownian motion of the particles and subsequently transforms this data into a particle size distribution function. As the size of the particles in suspension increases, the intensity of Brownian motion decreases, resulting in a prolonged correlation time and a broader particle size dispersion. It is important to note that DLS measurements can be influenced by a range of parameters, including particle concentration, shape and morphology, fluid composition, surfactants, temperature, and viscosity.

Dynamic Light Scattering (DLS) primarily quantifies time-varying fluctuations in scattered coherent light, which correspond to the decay of the autocorrelation function. These fluctuations originate from the diffusive motion of particles within the solution. From the mean diffusion coefficient (D) of the particles, the hydrodynamic diameter (dH) can be derived using the following Stokes–Einstein equation [18]:

(1) $$D=\frac{k_{B}T}{3\pi \eta_{0}d_{H}},$$

where k_B_ is the Boltzmann constant, T is the temperature and η_0_ is the viscosity of the fluid. The Zeta (ζ) potential was determined through experimental measurements of electrophoretic mobility (μ_e_) that represents the ratio of the particle’s velocity to the electric field causing its movement relative to the liquid bulk. To convert the electrophoretic mobility values into Zeta potential, the widely recognized Smoluchowski equation was employed, as expressed in the following equation:

(2) $$\mu_{e}=\frac{\varepsilon_{0}\varepsilon_{r\zeta }}{\eta },$$

where ε_0_ is the vacuum dielectric permittivity, ε_r_ is the relative dielectric permittivity and η is the dynamic viscosity of the liquid phase. DLS and ζ-potential measurements were performed on three independent samples (n = 3) to ensure reproducibility.

#### 2.4.4. Atomic Force Microscopy (AFM)

The Atomic Force Microscopy (AFM) examination on the chitosan microparticle samples provided an analysis of surface morphology and roughness as well as nano-mechanical properties of the samples. The AFM examination was conducted using a Bruker MultiMode 8. PeakForce Quantitative Nanomechanical Mapping (QNM) mode was employed to obtain high-resolution 2D and 3D topography images and roughness values, while Force Modulation mode was used to generate quantitative nanomechanical maps (stiffness and Young’s modulus). The scan area was 15 × 15 µm^2^ at a scan rate of 0.65 Hz. A RTESPA-300 cantilever (Bruker Corporation, Billerica, MA, USA) was employed, with a resonance frequency of approximately 300 kHz and a spring constant of 40 N/m. Data processing was carried out using Nanoscope Analysis software (version 1.5). The modulus data obtained in Force Modulation mode were fitted using the Hertzian (spherical) model.

#### 2.4.5. Determination of Encapsulation Efficiency (EE)

Chitosan microparticles were loaded with 30 mg of bovine serum albumin (BSA) during the synthesis process. The resulting BSA-loaded microparticles were then recovered by centrifugation and lyophilized. The amount of residual BSA in the supernatant was quantified using the Bradford protein assay. Based on the Bradford assay results, the encapsulation efficiency (%) was calculated according to the following equations [11,12]:

(3) $$EE\left(\%\right)=\frac{\text{Initial weight of BSA}-\text{Weight of BSA in supernatant}}{\text{Initial weight of BSA}}\times 100\%,$$

#### 2.4.6. In Vitro Drug Release Study

The cumulative release profile of BSA from chitosan microparticles was evaluated using a direct incubation method. A total of 10 mg of microparticles was suspended in 2 mL of PBS (pH 7.4) and incubated at 37 °C on a thermostated shaker (850 rpm). At predetermined time points (2 and 6 h, and 1, 2, 3, 7, 8, 9, 10, and 14 days), samples were centrifuged at 6000 rpm for 5 min and the supernatant was collected. The concentration of released BSA was determined using the Bradford protein assay and compared with the total amount of the initially encapsulated BSA in the microparticles [11,12,13].

In parallel, under the same incubation conditions, after 14 days, the microparticles were collected by centrifugation (6000 rpm, 5 min), washed with deionized water, and lyophilized for further analyses. The BSA release percentage was determined according to the following equation [19]:

(4) $$\text{BSA Release}\left(\%\right)=\frac{\text{Released BSA}}{\text{Initially encapsulated BSA}}\times 100\%,$$

All measurements were performed in triplicate (n = 3), providing a direct and reproducible evaluation of cumulative protein release and microparticle stability.

## 3. Results and Discussion

### 3.1. Chemical Structure and Functional Group Analysis

**Figure 2:**
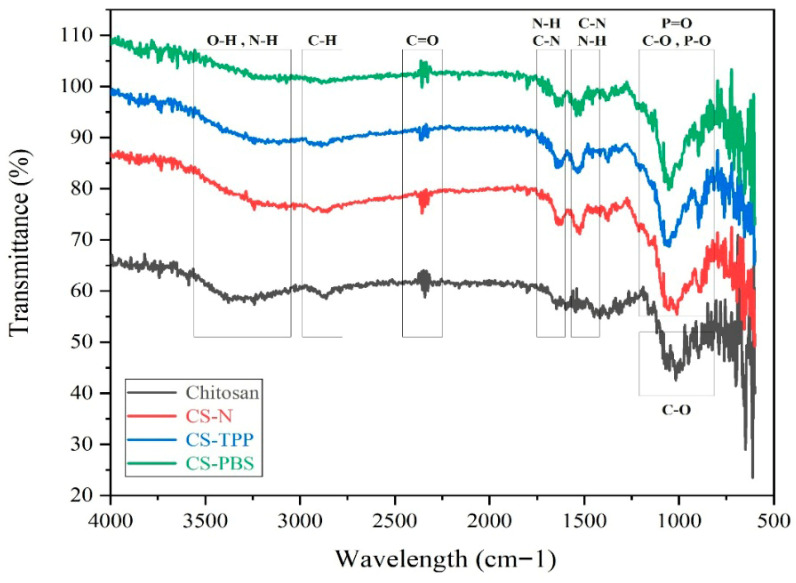
ATR spectra of native chitosan and synthesized microparticles (CS–N, CS–TPP, CS–PBS), highlighting functional group interactions and structural changes following ionic gelation and stabilization.

ATR spectra of native chitosan and the microparticle formulations CS-N, CS-TPP, and CS-PBS are presented in Figure 2. CS-PBS samples were extensively washed by repeated centrifugation, minimizing the contribution of residual buffer salts to the observed phosphate-related FTIR signals. All samples exhibit characteristic absorption bands of chitosan, confirming that the fundamental polymeric structure is retained after microparticle synthesis. These findings are in agreement with previous ATR-FTIR analyses of chitosan-based systems, where similar bands were reported as identifiers of the polysaccharide backbone [2,5]. A broad band in the 3400–3200 cm^−1^ region is attributed to the stretching vibrations of –OH and –NH_2_ groups, indicative of the polysaccharide backbone and primary amine functionalities. The absorption peaks at approximately 2920 and 2850 cm^−1^ correspond to asymmetric and symmetric C–H stretching, confirming the aliphatic nature of the chitosan chains. The distinct band around 1650 cm^−1^ is assigned to amide I (C=O stretching), while the shoulder near 1550 cm^−1^ corresponds to amide II (N–H bending and C–N stretching), reflecting molecular interactions and potential protonation effects during the ionic gelation process. In CS-TPP, where tripolyphosphate (TPP) was used as an additive, and in CS-PBS, which contains phosphate-buffered saline (PBS), additional peaks appear in the 1200–1250 cm^−1^ region, corresponding to P=O stretching, and in the 1020–1070 cm^−1^ range, attributed to P–O and C–O stretching.

The presence of these phosphate-related bands has also been observed in previous studies involving TPP- or phosphate-modified chitosan systems, supporting the occurrence of ionic interactions [3,4]. These features suggest successful ionic interactions between phosphate groups and the protonated amino groups of chitosan. These phosphate-associated bands are absent or significantly less intense in CS-N, which lacks any additive, confirming the role of phosphate-containing compounds in inducing molecular-level interactions. Moreover, the slight shifts and broadening of characteristic peaks in CS-TPP and CS-PBS compared to native chitosan indicate structural rearrangements and effective crosslinking. Such spectral changes are commonly attributed to molecular reorganization and crosslinking events, as reported in related ionic gelation studies [11]. Collectively, these observations validate the successful formation of chitosan microparticles via ionic gelation, with TPP and PBS introducing distinct chemical signatures identifiable through ATR spectroscopy.

### 3.2. Particle Size Characterization

**Figure 3:**
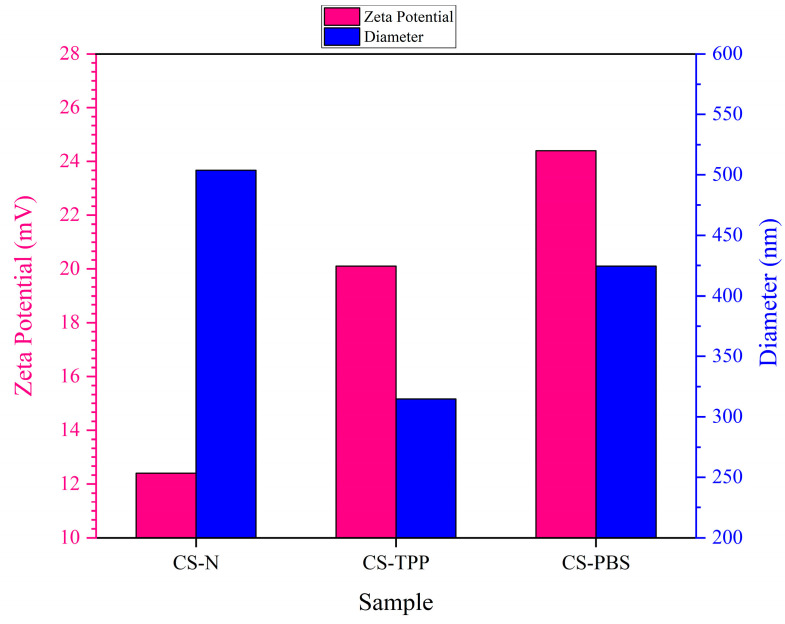
Effect of Additives on Particle Size and Zeta Potential of Chitosan Microparticles.

The particle size distribution and zeta potential of chitosan microparticles were evaluated using Dynamic Light Scattering (DLS) to assess the hydrodynamic diameter and surface charge characteristics of all three formulations: CS–N, CS–TPP, and CS–PBS. As illustrated in Figure 3 and summarized in Table 3, the inclusion of different modifiers—namely none (CS–N), tripolyphosphate (TPP; CS–TPP), and phosphate-buffered saline (PBS; CS–PBS)—had distinct effects on the physicochemical properties of the resulting particles, particularly in terms of colloidal stability and particle size.

In the absence of any additive (CS–N), chitosan microparticles exhibited a relatively large mean diameter of 503.8 nm and a moderate zeta potential of +12.4 mV, indicating limited electrostatic stabilization. The positive surface charge stems from the protonated amine groups of chitosan; however, without ionic crosslinking or buffering, these particles are prone to irregular growth and aggregation due to unbalanced charge distribution during synthesis [8,11]. When TPP was added as a multivalent anionic crosslinker, it formed strong ionic bridges with the positively charged amino groups of chitosan, yielding more compact and uniformly crosslinked microparticles. The resulting CS–TPP particles exhibited a smaller average size (314.9 nm) and a higher zeta potential (+20.1 mV), both of which reflect enhanced structural integrity and colloidal stability. These trends align well with previous reports demonstrating the stabilizing and size-reducing effects of TPP-mediated ionic gelation in chitosan systems [2,4].

In contrast, PBS acts as an isotonic buffer rather than a crosslinker. It does not directly bind to chitosan but helps maintain a stable ionic environment and pH during synthesis. CS–PBS microparticles showed a mean size of 424.5 nm and the highest zeta potential (+24.4 mV) among all formulations. The increased surface charge is attributed to effective ionic shielding and stabilization, which minimize aggregation through enhanced electrostatic repulsion [3]. Together, these findings underscore the importance of formulation additives in modulating chitosan microparticle characteristics. TPP enhances particle compactness and uniformity via ionic crosslinking, while PBS improves surface charge and dispersibility through electrostatic effects. These tunable colloidal properties are critical for biomedical applications, particularly in protein delivery and injectable scaffolds, where particle size and surface charge directly affect tissue interaction, diffusion behavior, and drug release kinetics [12,13].

### 3.3. Microstructure Characterization

**Figure 4:**
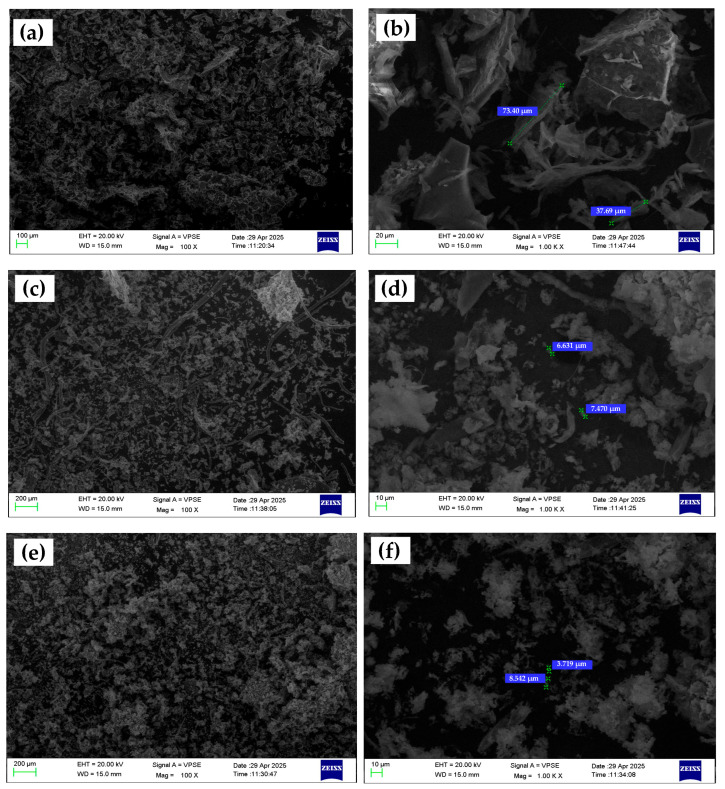
Representative SEM images of chitosan microparticles synthesized via ionic gelation under different formulation conditions. (**a**,**b**) CS–N (no additive), (**c**,**d**) CS–TPP, (**e**,**f**) CS–PBS. Left images show overall particle distribution; right images display higher magnification views with size annotations.

Scanning Electron Microscopy (SEM) was utilized to investigate the surface morphology, size, and dispersion characteristics of chitosan microparticles fabricated under three formulation conditions: without any additive (CS–N), with tripolyphosphate (CS–TPP), and with phosphate-buffered saline (CS–PBS). Representative low- and high-magnification SEM images (100× and 1000×, respectively) are shown in Figure 4. At low magnification (100×), the CS–N formulation exhibits large, irregular aggregates with poorly defined boundaries. The absence of a crosslinking or rate-controlling agent during ionic gelation leads to uncontrolled nucleation and particle growth, resulting in extensive coalescence and high polydispersity. High-magnification images (1000×) further reveal amorphous, non-spherical structures with rough, uneven surfaces and minimal inter-particle separation. These morphological features are consistent with DLS results showing a broad size distribution (503.8 nm) and a moderate zeta potential (+12.4 mV). The lack of electrostatic or steric stabilization during synthesis contributes to low colloidal stability and poor control over particle formation [14,15].

In contrast, the CS–TPP microparticles display a spherical and well-defined shape, with uniform size distribution visible even at low magnification. The images at 1000× magnification reveal smooth and compact surfaces, indicating efficient ionic crosslinking between TPP’s phosphate groups and the protonated amino groups of chitosan. These particles are more discrete and less prone to agglomeration, which correlates well with their smaller average size (314.9 nm) and higher zeta potential (+20.1 mV) obtained from DLS analysis. The use of TPP promotes controlled gelation kinetics, enhances particle integrity, and supports the formation of reproducible, monodisperse structures [17]. The CS–PBS formulation presents intermediate morphological characteristics. At 100×, particles are moderately dispersed with occasional clustering. High-magnification images show smoother surfaces and more regular shapes than CS–N, but they remain less compact and defined compared to CS–TPP particles. Since PBS lacks crosslinking functionality, its role is limited to buffering and ionic stabilization during the synthesis. Nevertheless, this buffering effect helps regulate particle formation, leading to improved size distribution (424.5 nm) and the highest zeta potential (+24.4 mV) among the three formulations. These findings suggest that PBS enhances electrostatic repulsion between particles through ionic shielding, though without contributing to crosslink-induced compaction [17,20].

It is also important to note that the particle sizes observed in SEM images were significantly larger than those measured via Dynamic Light Scattering (DLS). This discrepancy can be attributed to differences in sample preparation and measurement principles. While DLS assesses the hydrodynamic diameter of dispersed particles in solution, SEM analyzes dried, immobilized particles under vacuum, often after solvent evaporation and metal coating. The drying process tends to induce aggregation or flattening, particularly in soft polymeric materials like chitosan, resulting in larger or more variable observed dimensions [21,22,23]. In addition, the absence of stabilizing dispersants during SEM sample preparation may further promote coalescence, especially in non-crosslinked systems such as CS–N [24,25]. These findings are consistent with prior literature on polymer-based microparticles, which emphasizes the critical role of sample state (wet vs. dry) in determining particle size and morphology [26].

Overall, the SEM observations support the hypothesis that the inclusion of ionic modifiers significantly influences the morphology, uniformity, and compactness of chitosan microparticles. TPP facilitates well-defined crosslinked structures, while PBS contributes to colloidal stabilization without promoting significant morphological tightening. These microstructural differences are critical for tailoring microparticle performance in drug delivery systems.

### 3.4. Surface Topography and Roughness

**Figure 5:**
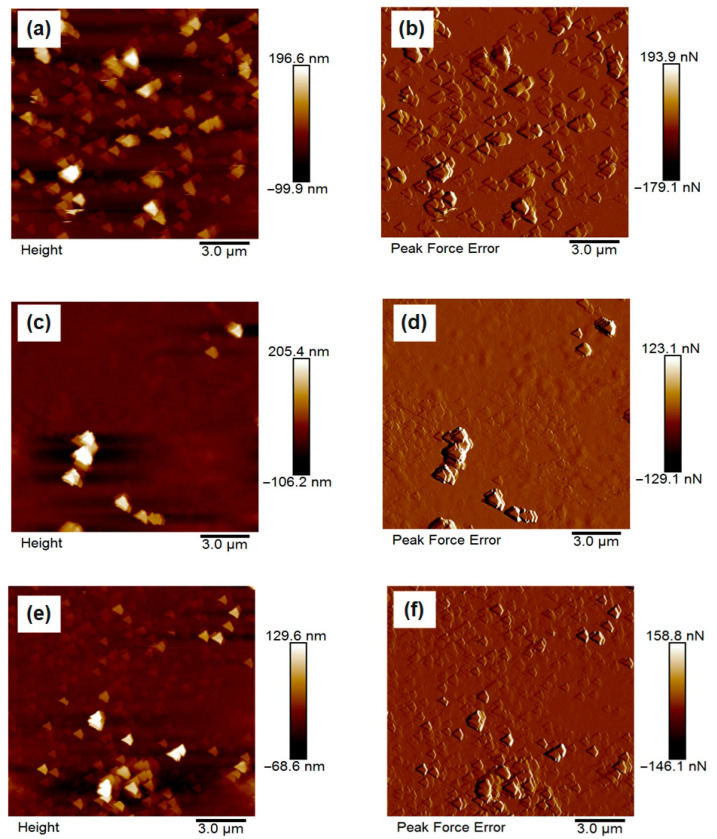
AFM topography images (2D and 3D) of chitosan microparticles synthesized with different additives: (**a**,**b**) CS–N (no additive), (**c**,**d**) CS–TPP (with tripolyphosphate), and (**e**,**f**) CS–PBS (with phosphate-buffered saline). Images highlight the influence of modifiers on particle surface morphology and compactness.

The surface morphology of the synthesized chitosan microparticles was investigated using atomic force microscopy (AFM), and representative 2D topographic images of the height and peak force error are presented in Figure 5a–f. The micrographs clearly demonstrate the influence of different formulation additives on the surface architecture and compactness of the particles.

In Figure 5a,b, corresponding to the unmodified chitosan microparticles (CS–N), the surface appears relatively coarse and heterogeneous, with pronounced aggregation of loosely bound microspheres. The particles display irregular shapes and lack structural compactness, which is indicative of limited stabilization during synthesis due to the absence of ionic crosslinkers or buffering agents. These observations align with previous findings that unmodified chitosan systems tend to form larger, more aggregated particles with rougher surfaces due to uncontrolled gelation and interparticle fusion [27,28]. In contrast, Figure 5c,d depict the morphology of CS–TPP particles, synthesized in the presence of tripolyphosphate. These samples exhibit notably smoother and more compact spherical particles with relatively uniform size and well-defined boundaries. The improved morphology is attributed to the ionic crosslinking between the phosphate groups of TPP and the protonated amino groups of chitosan, which facilitates the formation of stable, tightly packed microstructures [29,30]. Previous AFM studies have similarly shown that TPP-crosslinked chitosan particles exhibit reduced roughness and enhanced homogeneity [31]. Figure 5e,f, corresponding to CS–PBS, show particles with an intermediate surface profile. While their morphology is more uniform and less aggregated than CS–N, it remains slightly rougher and less compact than that of CS–TPP. This can be explained by the non-crosslinking but buffering role of PBS, which regulates pH and ionic strength during gelation, thus improving dispersion without inducing strong structural cohesion [32]. Overall, the AFM results confirm that the choice of additive plays a critical role in controlling surface morphology, and that ionic crosslinkers such as TPP are particularly effective in enhancing particle compactness and surface smoothness. These structural characteristics are essential for improving reproducibility and functional performance in biomedical applications [33,34,35].

Quantitative surface roughness analysis using AFM revealed that the surface characteristics of chitosan microparticles were strongly influenced by the type of modifying agent employed during synthesis. As presented in Table 4, CS–N, synthesized without any additive, exhibited the highest root mean square roughness (Rq = 11.84 nm), indicating pronounced surface irregularities and a higher degree of particle aggregation. This can be attributed to the lack of electrostatic stabilization, resulting in poorly controlled gelation and interparticle fusion. In contrast, CS–TPP, formulated with tripolyphosphate (TPP) as a multivalent ionic crosslinker, demonstrated the lowest roughness (Rq = 4.97 nm), suggesting the formation of compact and structurally uniform particles. This improvement is likely due to ionic interactions between the negatively charged phosphate groups in TPP and the protonated amino groups in chitosan, which enhance crosslinking density and morphological stability [4,8,11]. The CS–PBS formulation exhibited an intermediate roughness value (Rq = 5.97 nm), reflecting partial stabilization through modulation of ionic strength by phosphate-buffered saline. Although PBS does not induce crosslinking, its buffering capacity appears to facilitate more homogeneous particle dispersion during synthesis. These findings are consistent with previous studies reporting that increased crosslinking efficiency and particle compactness correlate with reduced surface roughness in chitosan-based delivery systems [11,17,21].

**Figure 6:**
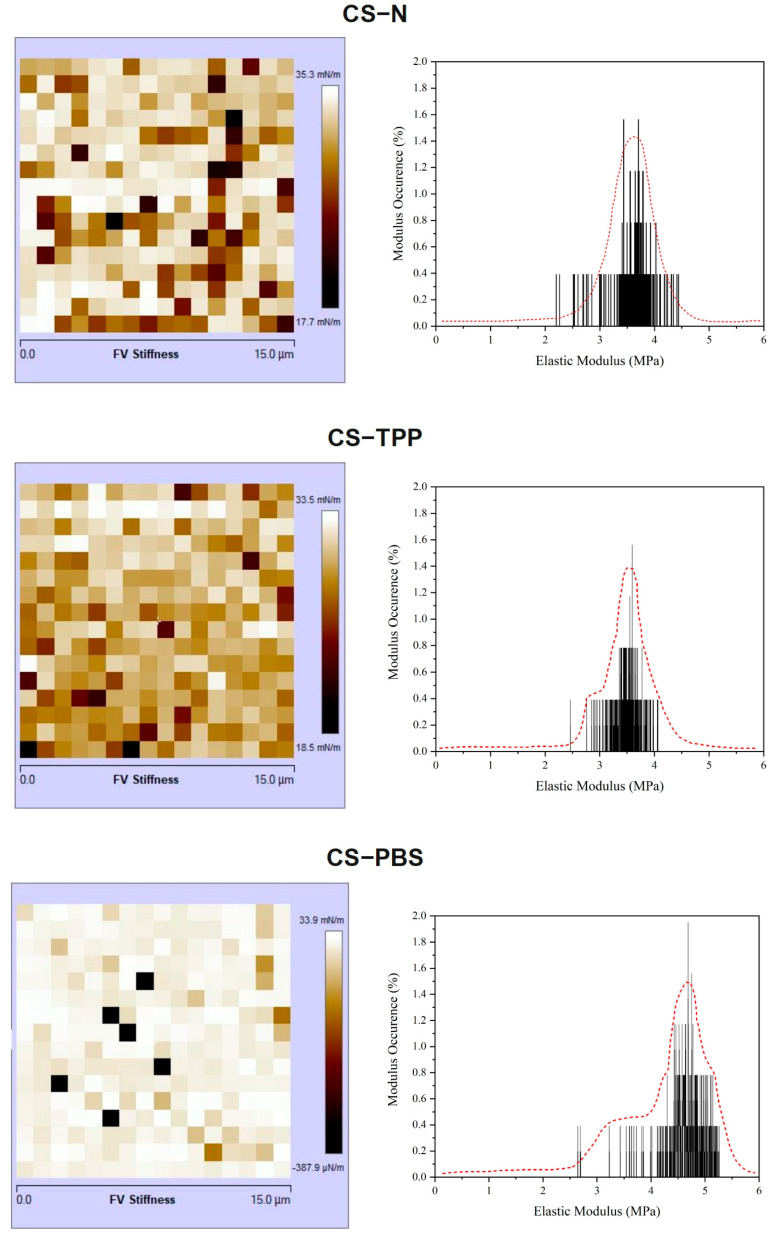
Nanomechanical characterization of chitosan microparticles using AFM. Left: stiffness maps (force volume) indicating surface heterogeneity across samples. Right: corresponding elastic modulus histograms showing distribution patterns. CS–N exhibits a broad and irregular modulus profile, CS–TPP shows a more uniform distribution due to crosslinking effects, and CS–PBS presents a bimodal distribution, consistent with partial ionic stabilization without structural consolidation.

Through atomic force microscopy (AFM), Force Modulation mode, additional information regarding the surface integrity and stiffness distribution of the chitosan microparticles was gained as seen in Figure 6. The CS–N sample had the most heterogeneous stiffness map, along with a broad distribution of elastic modulus values (centered around ~3.5 MPa) clearly indicating the most mechanically irregular surface, which was consistent with the rougher and less cohesive microstructure observed in both AFM and SEM images. In contrast, the CS–TPP microparticles displayed a more homogeneous stiffness profile, with a narrower modulus distribution (centered around ~3.7 MPa) identifying a uniform mechanical reinforcement. This consistent stiffness profile is attributed to ionic crosslinking resulting from the interaction between tripolyphosphate (TPP) and chitosan, where the protonated amino groups have interacted, leading to a less compliant, more compact network with reduced structural randomness and enhanced uniformity. Interestingly, the CS–PBS exhibited both the highest peak modulus (~4.5–4.8 MPa) and a bimodal elastic modulus distribution, suggesting the presence of distinct mechanical domains within the structure, comprising both stiff and soft regions. This duality is consistent with the knowledge that PBS is not acting as a crosslinker, but rather an ionic strength mediator of surface charges and has only partially stabilized the system, resulting in an inherently more stable particle but not necessarily a more consistently mechanically uniform particle. Given the mechanical results have an obvious relationship to the particle structural morphology and roughness studies it can be concluded that crosslinking with TPP not only resulted in more consistent structural uniformity, but defined the ease of mechanic behavior, while PBS had little more than a contribution to modulating surface charge and superficial compactness of the particle [8,17,32].

Although direct degradation studies were not performed, AFM nanomechanical measurements showed significant differences in stiffness distributions from CS-N, CS-TPP, and CS-PBS microparticles which may be indicative of possible particle stability. CS-TPP had a relatively consistent stiffness suggesting a uniform mechanical response exhibited across the particle group and is consistent with previous work associated uniform nanomechanical properties with better structural integrity and stability in chitosan nanoparticles [33,34]. CS-N had a very heterogeneous stiffness distribution suggesting that the mechanical behavior varied considerably and could lend itself to less predictable stability. The stiffness distribution in CS-PBS suggested a bimodal stiffness distribution suggesting there are at least two mechanically different subpopulations in the sample. Future studies will experimentally investigate the direct relationship between nanomechanical properties and potential particle degradation, building on these mechanistic insights.

### 3.5. Determination of Encapsulation Efficiency (EE)

**Figure 7:**
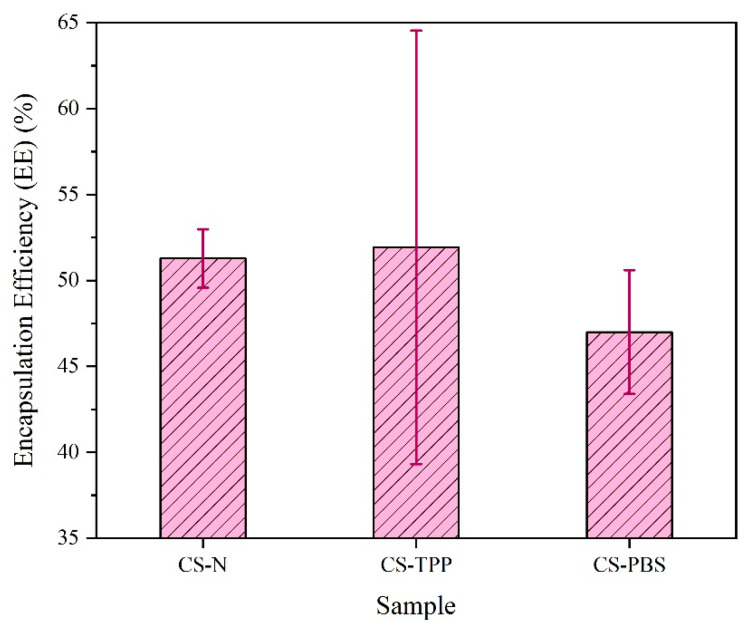
Encapsulation efficiency (%EE) of BSA in chitosan microparticles prepared without additive (CS–N), with tripolyphosphate as crosslinker (CS–TPP), and with phosphate-buffered saline (CS–PBS). CS–TPP exhibited the highest mean EE%, while CS–PBS showed a slightly reduced efficiency, likely due to the absence of crosslinking. Error bars represent standard deviation (n = 3).

The encapsulation efficiency (EE%) outcomes for BSA-loaded chitosan microparticles are presented in Figure 7, highlighting the effect of different modifiers—TPP and PBS—on protein entrapment. In the examined formulations, CS–TPP had the highest degree of EE% (51.93 ± 12.6%), which is only marginally greater than the unmodified formulation CS–N (51.28 ± 1.7%). In comparison, the PBS-modified sample (CS–PBS) resulted in a slightly lower encapsulation efficiency (47.0 ± 3.6%). The improved EE% in CS–TPP was likely due to the ionic crosslinking action of tripolyphosphate (TPP), forming electrostatic bridges with the protonated amino groups of chitosan. This facilitates a polymeric way of density involving facilitating BSA physically during microparticle formation. Furthermore, the high standard deviation indicates the potential for inconsistency of data across batches, and this may not have been due to differences in methodology in crosslinking or even particle structure. In contrast, CS–PBS yields lower EE% as there was no crosslinking mechanism. Not to mention, while PBS creates ionic strength balance, thus stabilizing the colloidal environment, it lacks potent interaction with chitosan to form a compact matrix, resulting in more BSA remaining in the supernatant during synthesis.

Additionally, unmodified CS–N will produce a similar EE% as CS–TPP but with considerably less variability, which might indicate that a more uniform particle was formed without the addition of modifiers based on BSA being entrapped by simple electrostatic interactions only without having to establish a complex network. In conclusion, the results demonstrate the role formulation had with the addition of TPP or PBS buffer on the variability of EE%. In this case, TPP improved protein entrapping as a result of ionic crosslinking, but at the cost of variability, whereas PBS’s effect seems negligible with only limited impact on EE%, highlighting the role of crosslinking density and polymer–drug interactions in chitosan delivery systems. These results support earlier studies showing that TPP supports protein entrapment via ionic crosslinking with the protonated amine groups of chitosan. TPP results in better encapsulation efficiency [4,22]. In addition, the observed relatively low EE of the PBS formulation may be due to a lack of ionic gelation as mentioned by Grenha et al. [27], specifically for protein-loaded chitosan systems.

### 3.6. In Vitro Drug Release Study

**Figure 8:**
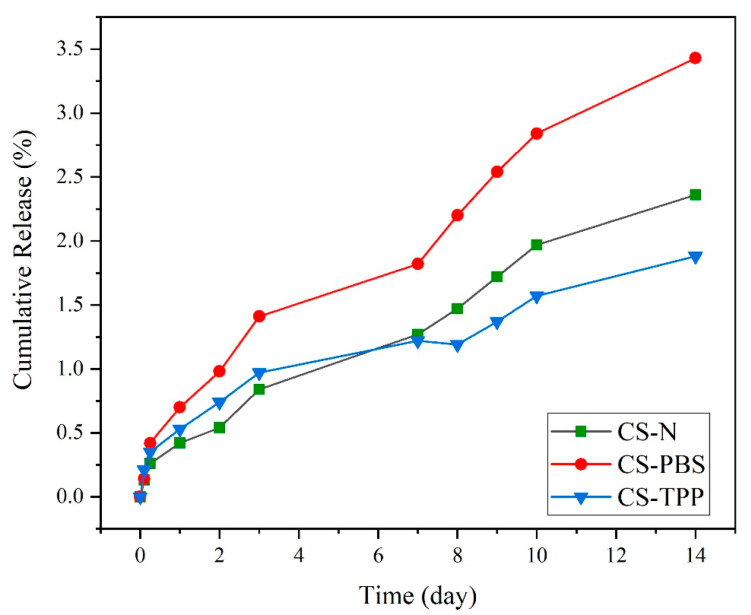
In vitro cumulative BSA release (%) for chitosan microparticles (CS–N, CS–TPP, and CS–PBS) over 14 days under physiological conditions (PBS, pH 7.4, 37 °C). BSA release was monitored using the Bradford assay.

The release experiment of BSA from chitosan microparticles over 14 days (Figure 8) shows important discrepancies in structural stability based on the presence and the type of modifying agent. The experiment took place in physiological conditions (PBS, pH 7.4, 37 °C) differences between formulations were observed.

The limited BSA release experienced (<3.5% over 14 days) suggests, rather than hydrolytic degradation of chitosan in the specific conditions of this study, a drug release mechanism mainly following passive diffusion, driven by water penetration into the polymeric network and the concentration gradient in the absence of enzymatic activity. In contrast, in enzyme-responsive systems, such as those involving lysozyme-mediated cleavage of chitosan, a dual contribution of diffusion and enzymatic degradation can be observed. Indeed, in these systems, the progressive enzymatic hydrolysis of glycosidic bonds accelerates matrix erosion and facilitates a more dynamic and often faster release compared to purely diffusion-based systems. Moreover, enzyme-triggered release is strongly affected by intrinsic polymer characteristics (degree of acetylation, molecular weight, and crosslinking density) as well as by physiological conditions (enzyme concentration, pH), thereby offering more complex and physiologically relevant release profiles than passive diffusion alone [12,36,37].

The unmodified formulation, CS–N, appeared to show moderate release over time and by day 14 had approximately 2.36% BSA release. The loss of structural integrity was expected because of the lack of stabilizing interactions, which would have prevented the entry of water into a loosely aggregated structure and would have preserved the mechanical strength of the material. Without crosslinking occurring, weak interactions existed between polymer chains, allowing for moderate but constant loss of protein over time. In contrast, CS-PBS exhibited the highest degree of release out of the three formulations, with a cumulative protein release of 3.43% after 14 days. While PBS does provide better dispersion, buffered ionic environment at the start of synthesis, there was no real chemical during the synthesis process or ionically crosslinking with the chains of chitosan. While the particles’ macroscopic appearance may look more homogenous to the initial dispersion (refer to SEM and AFM) the CS—PBS formulation remains less dense, and thus, liable to hydrolysis. This corresponds with our findings of dual-peak stiffness distribution (the stiffness measurement for this example observed a small increase in surface roughness from AFM), and therefore some form of partial stabilization with no further structural change.

In comparison, CS–TPP had the slowest release and the highest structural stability, with only 1.88% protein release after 14 days. The increased resistance to release was due to the ionic crosslinking of TPP (tripolyphosphate), creating strong electrostatic links between the negatively charged phosphate groups and between the protonated amino groups of chitosan. ATR-FTIR analysis provides further evidence for this interaction, given the emergence of characteristic phosphate bands (~1250 and 1070 cm^−1^). This interaction further develops a denser polymeric network, which hinders water penetration and delays hydrolytic cleavage. The CS–TPP also shows lower surface roughness, a narrower modulus distribution, and higher encapsulation efficiency that build the evidence for well-ordered, compact particles. Overall, the in vitro drug release data correlates very well with the structural and physicochemical data acquired by AFM, SEM, ATR, and EE. The degree of crosslinking, and how compact the matrix internally is, which depend on the inclusion of TPP, clearly dictate release behavior. The findings reported here emphasize the importance of crosslinking during design processes for the development of drug delivery systems. Sustained drug release necessitates a carrier that retains its physical and chemical stability over a long enough period, and that degrades slowly enough to allow for sustained delivery of the payload. The TPP-crosslinked microparticles represent the ideal characteristics needed for this application: reduced degradation, greater structural integrity, higher drug retention. Other publications have reported similar findings, demonstrating that chitosan–TPP systems are capable of prolonging the material performance of systems, and decreasing burst release due to the stronger polymeric networks they form [4,21,38]. The reported 14-day cumulative BSA release of 1.88–3.43% corresponds to the above-described procedure. This method provides a direct, quantitative assessment of microparticle stability under incubation conditions.

Literature indicates that release kinetics depend significantly on environmental conditions and the presence of specific enzymes. Our microparticles were tested under physiological conditions (PBS, pH 7.4, 37 °C), showing formulation-dependent BSA loss: CS-PBS (highest), CS-N (moderate), and CS-TPP (lowest). In contrast, enzymatic degradation involves specific enzymes like lysozyme that cleave glycosidic bonds in chitosan, resulting in accelerated polymer chain scission. The superior stability of CS-TPP demonstrates that crosslinking density is a critical factor controlling drug release rates under physiological conditions. These findings emphasize the importance of formulation design in developing chitosan-based delivery systems for biomedical applications [37].

## 4. Conclusions and Future Outlooks

The present study produced chitosan microparticles using ionic gelation in a rate-controlled environment, where ionic modifiers were used (tripolyphosphate, TPP, or phosphate-buffered saline, PBS), and in the absence of additives. In general, the structure, surface morphology, and physicochemical properties were dependent on the type of additive used during synthesis. Characterization by ATR–FTIR indicated that the primary chitosan backbone remained intact, but there were phosphate-associated interactions present with the TPP- and PBS-modified formulations. Scanning electron microscopy and atomic force microscopy indicated that TPP facilitated the formation of denser, more compact particles that had smoother surfaces (less roughness) than those with PBS or non-modified chitosan microparticles, which provided moderate stabilization, but did not lead to ionic crosslinking. The micro-mechanical maps indicated that the CS−TPP formulation had the narrowest mechanical distribution and implied a more uniformly structured internal chitosan, which was also supported by the stiffness, modulus, and roughness maps. The quantitative results from dynamic light scattering also supported this description, given that TPP lowered particle size and yielded higher zeta potential, and suggested that TPP was a suitable ionic modifier to confer a colloidally stable microparticle system with maintained small size. The encapsulation efficiency of BSA was similar for CS–N and CS–TPP, but was lower in CS–PBS. The drug release profiles were sustained for all formulations, with CS–TPP showing comparatively slower release over time and improved retention, likely due to TPP’s strong ionic crosslinking potential. PBS-modified particles were much more stable than native particles at the start, but experienced higher release in the long run because there was no inter-chain binding. Overall, the findings suggest that formulation strategies involving ionic crosslinkers such as TPP can vastly improve the mechanical strength and stability of chitosan microparticles, as well as the retention of therapeutic molecules. This study offers insightful contributions toward the further development of chitosan microparticles in relation to tunable drug delivery, drugs retention and controlled-delivery applications. These findings suggest potential for chitosan microparticles to be used in drug delivery and tissue engineering applications, where tunable and sustained release are paramount to therapeutic efficacy. Albeit direct degradation studies were not performed, stiffness differences documented with CS–N, CS–TPP, and CS–PBS suggest potential implications for particle stability, consistent with previous reports. Future work will experimentally look at this relationship.

The chitosan microparticles fabricated in this work are highly viable biomedical products considering their tunable physicochemical properties, simple manufacturing method, and excellent biocompatibility. The chitosan structure, coupled with the ability to modify the surface of the microparticles, would allow us to make microparticles for targeted delivery and stimuli responsive release, such as pH or enzyme-sensitive delivery systems. In addition, it would be possible to include imaging agents, such as magnetic nanoparticles or fluorescence indicators with therapeutic agent-loaded drug, and the microparticles could be applied for diagnosis and therapy in the treatment of diseases, like cancer, chronic infections, and various inflammatory diseases.

## Figures and Tables

**Table 1:** Description of Chitosan Microparticle Samples with Stabilizer Usage and Preparation Methods.

Sample Code	Modifier Type	Description/Role
CS-N	None	No additive; ionic gelation occurs without stabilizers, leading to irregular particle formation and aggregation
CS–TPP	TPP (ionic crosslinker)	TPP facilitates electrostatic crosslinking between chitosan chains, reducing aggregation
CS–PBS	PBS (buffer stabilizer)	PBS controls ionic strength and pH to improve particle dispersion and stability

**Table 2:** Malvern Zetasizer Nano ZS spectroscopy.

Parameter/Feature	Specification/Value
Temperature control range	0 °C–90 °C ± 0.1 °C
Laser	4 mW, 633 nm
Correlator	25 ns–8000 s, max 4000 channels
Parameter measured	Particle size, zeta potential
**Particle size measurements**	
Absolute sensitivity (Toluene kcps)	150
Range	0.3 nm–10 µm
Min. sample volume	12 µL
Max concentration	40% *w*/*v*
Measurement angles	13° + 173°
**Zeta potential measurements**	
Zeta potential range	>±500 mV
Mobility range	>±20 µ·cm/V.s
Max. sample concentration	40% *w*/*v*
Max. sample conductivity	200 mS/cm
Signal processing	M3-PALS

**Table 3:** Zeta Potential and Size Characteristics of Chitosan Microparticles Formulated with and without Additives.

Sample	Diameter (nm)	Zeta Potential (mV)	PDI	Effect of Modifier
CS-N	503.8 ± 20.0	12.4 ± 1.2	0.388	Moderate charge, large size due to aggregation
CS–TPP	314.9 ± 12.0	20.1 ± 1.0	0.298	Enhanced charge, compact and smaller particles
CS–PBS	424.5 ± 15.0	24.4 ± 1.5	0.322	Strong surface charge, moderate size

**Table 4:** Roughness Values of Chitosan Microparticles Formulated with and without Additives.

Sample Code	Image Rq (nm)	Image Ra (nm)	Mean Roughness Rq (nm)
CS-N	12.6	9.64	11.84
CS–TPP	6.58	5.01	4.97
CS–PBS	7.03	5.04	5.97

## Data Availability

The original contributions presented in the study are included in the article, further inquiries can be directed to the corresponding authors.
